# Longer storage duration of red blood cells is associated with an increased risk of acute lung injury in patients with sepsis

**DOI:** 10.1186/2110-5820-3-33

**Published:** 2013-09-24

**Authors:** David R Janz, Zhiguo Zhao, Tatsuki Koyama, Addison K May, Gordon R Bernard, Julie A Bastarache, Pampee P Young, Lorraine B Ware

**Affiliations:** 1 Department of Medicine, Division of Allergy, Pulmonary, and Critical Care Medicine, Vanderbilt University School of Medicine, 1161 21st Avenue South, T-1218 Medical Center North, Nashville, TN 37232, USA; 2 Department of Biostatistics, Vanderbilt University, Nashville, TN, USA; 3 Department of Biostatistics, Vanderbilt School of Medicine, Nashville, TN, USA; 4 Department of Pathology, Microbiology and Immunology, Vanderbilt University School of Medicine, Nashville, TN, USA

**Keywords:** Acute respiratory distress syndrome, Transfusion-related acute lung injury, Red blood cell storage lesion

## Abstract

**Background:**

The storage duration of red blood cells transfused to critically ill patients is associated with increased morbidity and mortality. Whether the association exists between storage duration of red blood cells transfused to patients with sepsis and the risk of developing ALI/ARDS is unknown. We aimed to determine the association of the storage duration of red blood cells transfused to patients with sepsis and risk of developing acute lung injury in the subsequent 96 hours, with comparator trauma and nonsepsis/nontrauma groups.

**Methods:**

We conducted a retrospective observational study of 96 transfused, critically ill patients with sepsis, 176 transfused, critically ill patients with traumatic injury, and 125 transfused, critically ill nontrauma, nonsepsis patients. The primary outcome was the development of ALI/ARDS up to 96 hours after transfusion.

**Results:**

In 96 patients with sepsis, 49 (51%) patients developed ALI/ARDS. The median storage duration of transfused blood in the ALI/ARDS group was greater (24.5 days, interquartile range (IQR) 20–31) compared with the patients who did not develop ALI/ARDS (21 days, IQR 15–27, *p* = 0.018). Longer median storage duration was independently associated with an increased risk of developing ALI/ARDS in the subsequent 4 days (odds ratio 1.8, *p* = 0.028). The same association was not seen in the trauma or nonsepsis, nontrauma patients.

**Conclusions:**

Transfusion of blood with longer median storage duration to patients with sepsis is associated with a higher risk of developing ALI up to 4 days after transfusion. This same association is not seen in other critically ill patient populations.

## Background

Anemia is a common problem in critically ill patients and is ubiquitous in those remaining in an ICU for more than 2 days. The treatment of anemia in the ICU includes transfusion of red blood cells (RBCs) and may be used in as many as 40% of patients [1-3]. The practice of RBC transfusion to critically ill patients has recently come under scrutiny. Data have emerged describing the potential negative impact that RBC transfusion may have on outcomes, including increased ICU length of stay [4], nosocomial infections [5], multiorgan failure [6], acute lung injury [7-11], and mortality [2,12,13]. The pathophysiology underlying these adverse outcomes is not clear, but a growing body of data suggests a relationship with the storage duration of RBCs. With longer storage duration, there is an accumulation in the stored unit of biologically active lipids able to prime neutrophils [14,15], proinflammatory cytokines, such as IL-1, IL-8, TNF-α [16], procoagulant microparticles [17], and oxidants, including free hemoglobin and isoprostanes [18]. Consequently, longer storage duration of RBCs has been associated with some of the same adverse outcomes in humans, including multiorgan failure and increased risk of mortality [19-25], along with acute lung injury in animals [26-29]. However, the potential effect of storage duration of RBCs on the development of ALI in at-risk humans has not been systematically investigated nor is it known whether the risk of ALI related to storage duration of RBCs is consistent across different critically ill patient populations.

Based on the theoretical “two-hit hypothesis” for the development of transfusion-related ALI wherein neutrophils are initially primed by a risk factor (i.e., sepsis) and then activated by components of transfused blood [14,30], patients with sepsis who are exposed to RBCs may be a population at increased risk for ALI after transfusion with blood of a long storage duration. We performed a retrospective observational study to test the hypothesis that the storage duration of transfused blood is associated with an increased risk of developing ALI up to 96 hours after exposure in patients with sepsis. For comparison, we conducted similar analyses in patients with severe trauma and critically ill patients without trauma or sepsis.

## Methods

### Study design and patient population

The study population consisted of 1,334 patients who were enrolled prospectively in the Validating Acute Lung Injury Markers for Diagnosis (VALID) study [31-34] between January 1, 2008 and December 31, 2010. Although patients were enrolled in the VALID study beginning in 2006, electronically accessible blood bank records were not available prior to January 1, 2008. The VALID study aims to identify and validate plasma biomarkers for the diagnosis of ALI and includes patients who are ≥18 years old admitted to the Vanderbilt University Medical Center medical, surgical, cardiovascular, or trauma ICUs and who remain there for at least 2 days. All patients were enrolled on the morning of ICU day 2. A detailed description of study procedures has been published elsewhere [31,32]. All patients were phenotyped daily for ALI/ARDS by two study physicians using the AECC definitions [35] and daily review of radiographs, arterial blood gases, and the medical record.

**Figure 1:**
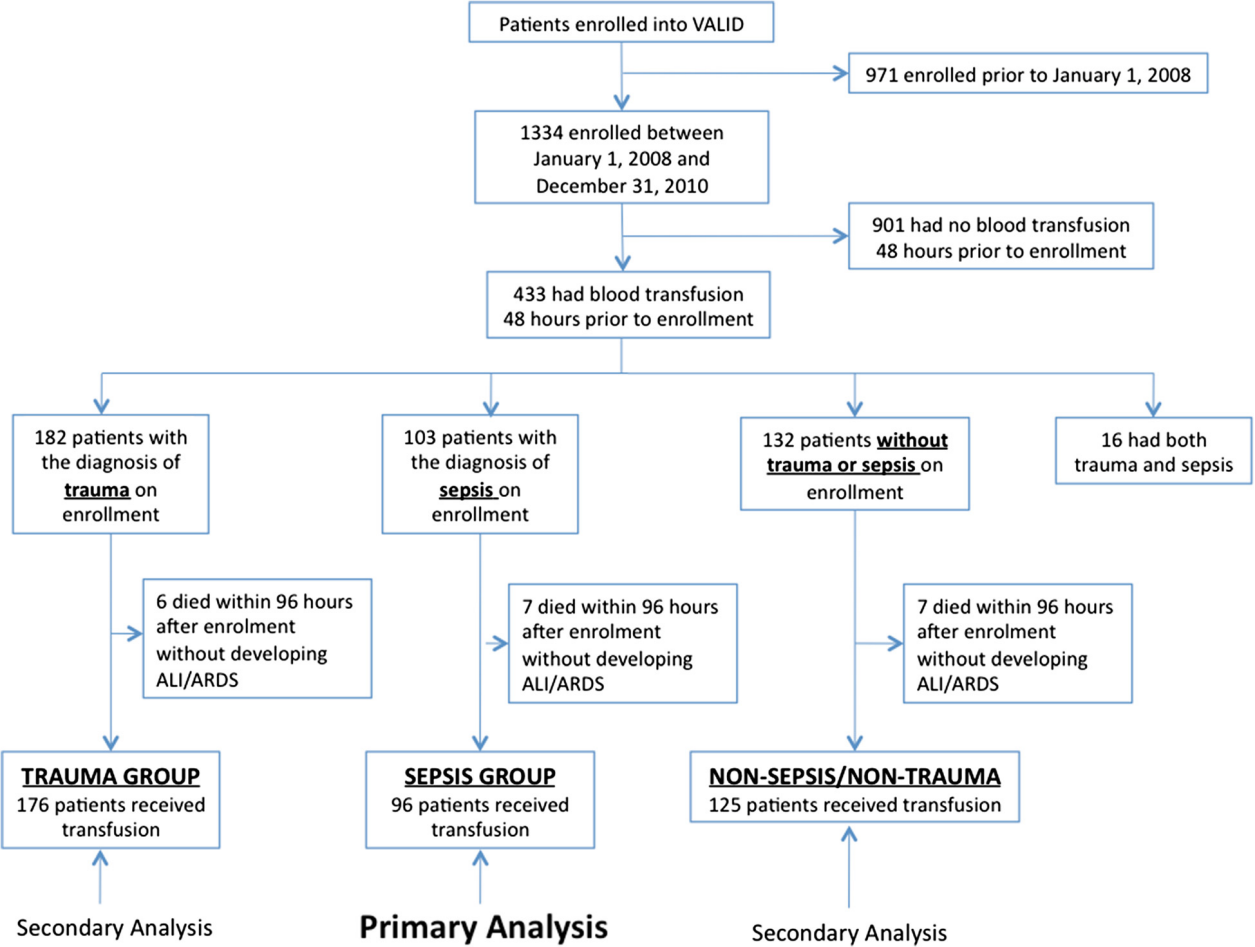
Flow diagram of patients included in each analysis.

For the 1,334 patients enrolled in VALID during the study window and after approval by the Vanderbilt University Institutional Review Board (IRB# 110506), we retrospectively searched their blood bank records to determine whether patients had received a RBC transfusion during the 48 hours before VALID study enrollment. For the 433 patients who had RBC transfusions during this interval, we recorded specific data regarding each unit of RBCs transfused including the date of transfusion and the date of collection from the donor. Storage duration of blood was calculated as the time from donation to transfusion. For inclusion in the sepsis group, patients had to have a diagnosis of sepsis [36] on enrollment into VALID and also have had received transfusion with RBCs in the 48 hours before enrollment. Inclusion in the trauma group required severe traumatic injury leading to ICU admission and receipt of a blood transfusion with RBCs in the 48 hours before enrollment (Figure 1). Patients who were admitted to the ICU with severe traumatic injury who also met criteria for severe sepsis at enrollment (n = 16) were excluded from the analysis. All other critically ill patients who received RBCs in the 48 hours before enrollment were classified as nonsepsis, nontrauma. The window for classifying patients with the presence or absence of ALI/ARDS was 96 hours after enrollment/transfusion. Because some patients developed ALI/ARDS 1 day or more after enrollment and the fixed RBC exposure period of 48 hours before enrollment may not account for all blood exposure for this group, we conducted an additional sensitivity analysis by excluding patients who developed ALI/ARDS 1 day or more after enrollment.

### Description of the storage of blood

All RBCs that were transfused were stored in the Vanderbilt University Medical Center’s (VUMC) Blood Bank according to guidelines established by the Food and Drug Administration. Specifically, these units of blood were leukoreduced using the Pall® Cell Processing Set (Ann Arbor, MI) within 24 hours of collection and before storage. The collection center tests 1% of all filter types collected and manufactured. The quality control is monitored each month and meets the American Academy of Blood Banks requirement of 95% of products tested pass the leukoreduction requirement. RBCs were not washed nor were any antioxidant solutions added. The maximum storage duration of RBCs at VUMC is 42 days.

### Statistical analysis

We selected, *a priori,* patients with sepsis, as defined by the consensus definition [36], on enrollment as our population of interest. As the majority (88.5%) of ALI/ARDS in this group occurred on the day of enrollment into VALID and because the requirement for transfusion-related acute lung injury to develop within 6 hours of transfusion [30] may be too restrictive [37,38], we set an exposure period of 48 hours before VALID enrollment for the analysis. The primary outcome was development of ALI/ARDS during the 96 hours after this exposure period. The same exposure period and outcome windows were applied to the trauma and nontrauma nonsepsis patients.

Transfusion data were summarized using the median storage duration of all RBCs transfused in the 48 hours before enrollment into VALID. We also analyzed the maximum storage duration, defined as the storage duration of the unit of RBCs with the longest storage duration transfused to a patient during the exposure window.

Wilcoxon rank-sum tests were used to compare storage durations between patients with and without acute lung injury and Pearson χ^2^ tests were used to compare categorical variables. Logistic regression models were developed to investigate the associations between the potential risk factors and the primary outcome of ALI/ARDS. Multiple imputation methods were used in the regression models to account for missing data [39]. Penalized maximum likelihood estimation (PMLE) was used to account for the potential impact of model overfitting [40]. A 300-iteration bootstrapping was used to validate the PMLE models. All analyses were performed using R 2.12.1 (R Development Core Team, Vienna, Austria). All tests were two-tailed, and a *P* value of 0.05 or less was considered significant.

## Results

### Sepsis patients

**Figure 2:**
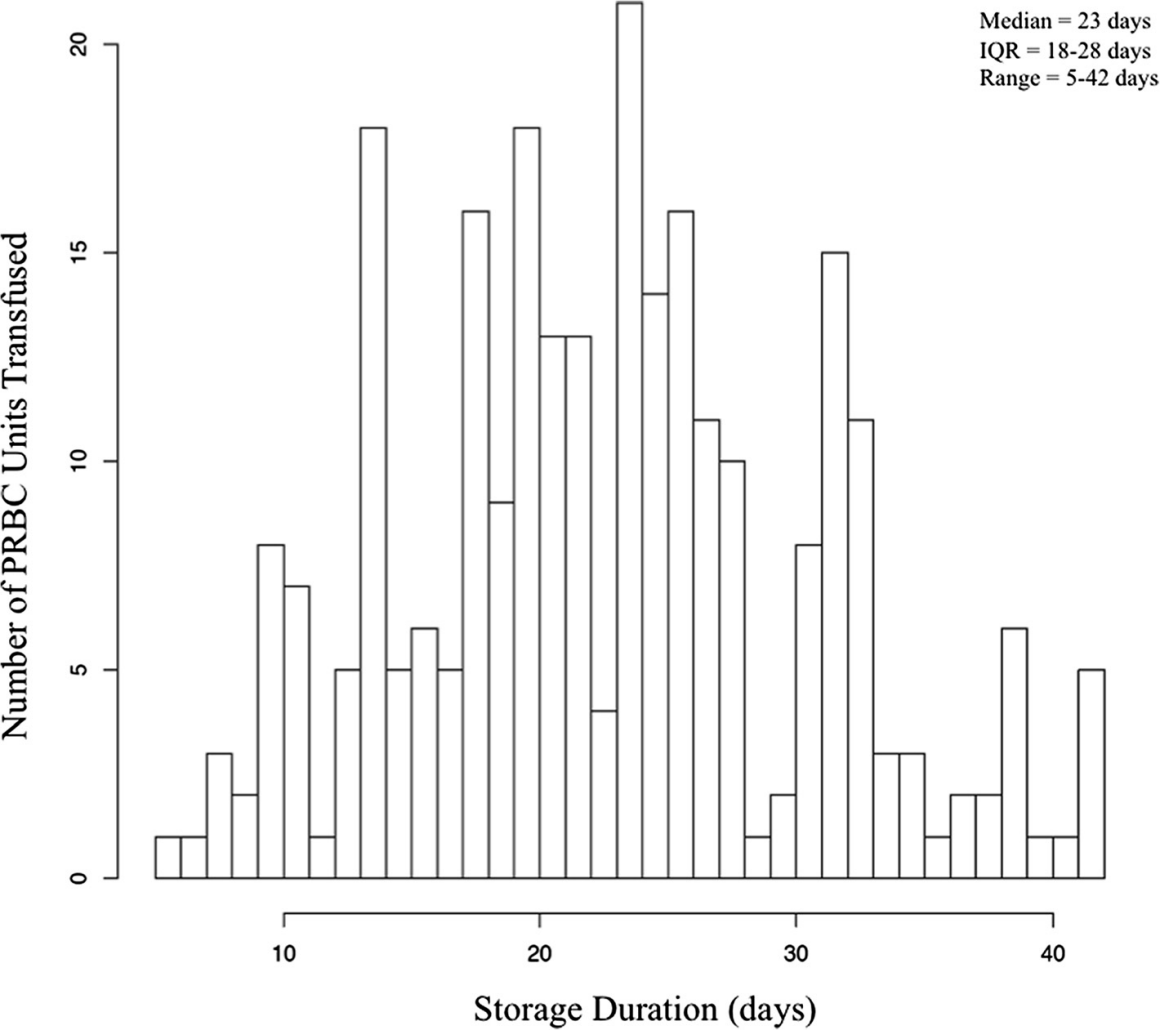
Distribution of storage duration of packed red blood cells transfused to patients with sepsis.

Of the 433 patients enrolled in the VALID study who had RBC transfusions during the 48 hours before enrollment into the VALID study, 96 met diagnostic criteria for sepsis on enrollment and comprised the study population for the primary analysis (Table 1). The median storage duration of RBCs transfused to these 96 patients was 23 days, interquartile range (IQR) 18–28 (Figure 2).

**Table 1:** Baseline characteristics of patients with sepsis

**Characteristic**	**ALI/ARDS (n = 49)**	**No ALI/ARDS (n = 47)**	***p *value**
Age	53 (45, 62)	61 (52, 66.5)	0.021^a^
Female gender	46.9% (23)	42.6% (20)	0.666^b^
Current smoker	27.1% (13)	31.9% (15)	0.606^b^
ICU days	8 (5, 14)	6 (4, 11)	0.118^a^
Ventilator-free days	18 (1, 24)	25 (7.5, 28.0)	0.022^a^
Hospital days	17 (11, 26)	14 (9.5, 26.5)	0.325^a^
Baseline APACHE II score	29 (24, 32)	29 (22.5, 35)	0.751^a^
Blood median storage days	24.5 (20, 31)	21 (15, 26.8)	0.018^a^
Blood maximum storage days	26 (22, 33)	24 (17, 28)	0.01^a^
Number of Units Transfused	2 (2, 3)	2 (2, 4)	0.303^a^

Data given as median (25^th^ percentile, 75^th^ percentile) or percent (frequency) of patients.

^a^Wilcoxon test; ^b^Pearson test.

In these transfused patients with sepsis, 49 (51%) developed ALI/ARDS in the 96 hours after transfusion. The median storage duration of RBCs transfused in the patients who developed ALI/ARDS was significantly longer (25 days, IQR 20–31) than the storage duration in those who did not develop ALI/ARDS (21 days, IQR 15–27, *p* = 0.018). Similarly, the maximum storage duration of RBCs transfused to the patients who developed ALI/ARDS was significantly longer (26 days, IQR 22–33) than the maximum storage duration of RBCs transfused to patients who did not develop ALI/ARDS (24 days, IQR 17–28, *p* = 0.01). The total number of units transfused during the exposure window did not differ between patients who developed ALI/ARDS (2 units, IQR 2–3) and those who did not (2 units, IQR 2–4, *p* = 0.303).

**Figure 3:**
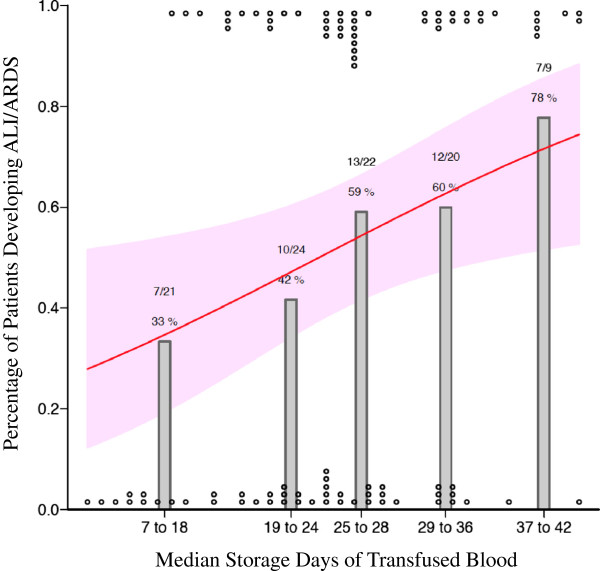
**Percentage of sepsis patients developing ALI/ARDS in the 4 days after transfusion and median storage duration of transfused red blood cells.** Grey bars represent the percentage of patients with sepsis (n = 96) who developed ALI/ARDS during the 96 hours after transfusion. Circles at the top and bottom represent cases and controls placed at their respective median age of transfused blood. The black line and grey confidence band represent a fitted line showing the probability of developing ALI/ARDS derived from the multivariable logistic regression and the corresponding 95% confidence interval.

After controlling for known risk factors for the development of ALI/ARDS, such as age, APACHE II score on enrollment, smoking status, and number of units transfused, there was a significant increase in the risk of developing ALI/ARDS with exposure to blood of a longer median storage duration (odds ratio (OR) 1.8 for the 75^th^ vs. 25^th^ percentile of storage duration, 95% CI 1.07-3.04, *p* = 0.028; Table 2; Figure 3) and longer maximum storage duration (OR 2.0 for the 75^th^ vs. 25^th^ percentile of storage duration, 95% CI 1.12-3.58, *p* = 0.02; Table 3). The sensitivity analysis excluding 6 patients who developed ALI/ARDS 1 day or more after enrollment showed similar results, with the odds ratios for median and maximum storage duration remaining significant (OR 2.09, *p* = 0.018 and OR 2.29, *p* = 0.018, respectively).

**Table 2:** Median storage days and risk of ALI/ARDS in patients with sepsis

**Characteristics**	**Odds ratio**	**95% CI**	***p *value**
Median storage days (28 vs. 18)	1.8	1.07-3.04	0.028
Unit of transfused blood (4 vs. 2)	0.93	0.57-1.52	0.765
Age (65 vs. 47 yr)	0.62	0.37-1.04	0.069
Baseline APACHE II score (33 vs. 23)	0.87	0.51-1.5	0.612
Current smoker (yes vs. no)	0.93	0.42-2.07	0.863

Odds ratio for median storage days is the odds of developing ALI/ARDS in the 96 hours after transfusion for the patients receiving RBCs in the 75^th^ compared with 25^th^ percentile of storage duration.

**Table 3:** Maximum storage days and risk of ALI/ARDS in patients with sepsis

**Characteristics**	**Odds ratio**	**95% CI**	***p *value**
Maximum storage days (32 vs. 20)	2	1.12-3.58	0.02
Unit of transfused blood (4 vs. 2)	0.88	0.54-1.45	0.626
Age (65 vs. 47 yr)	0.64	0.38-1.07	0.089
Baseline APACHE II score (33 vs. 23)	0.89	0.52-1.54	0.676
Current smoker (yes vs. no)	0.86	0.39-1.91	0.717

Odds ratio for maximum storage days is the odds of developing ALI/ARDS in the 96 hours after transfusion for the patients receiving RBCs in the 75^th^ compared with 25^th^ percentile of maximum storage duration, defined as the unit of RBCs of the longest storage duration received by a patient.

### Trauma patients

Of the 433 patients who received RBC transfusions 48 hours before enrollment in the VALID study, 176 patients were admitted to an ICU as a result of a traumatic injury, which defined the study population for this secondary analysis (Table 4).

**Table 4:** Baseline characteristics of the trauma cohort

**Characteristic**	**ALI/ARDS**	**No ALI/ARDS**	***p *value**
	**(n = 80)**	**(n = 96)**	
Age (yr)	51 (36, 63)	46 (29, 59)	0.094^a^
Female gender	28.8% (23)	24.0% (23)	0.471^b^
Current smoker	46.3% (31)	46.5% (40)	0.976^b^
ICU days	12 (7, 16)	8 (5, 13)	0.014^a^
Ventilator-free days	17 ( 12, 22)	21 (16, 24)	<0.001^a^
Hospital days	16 (11, 24)	15 (10, 23)	0.281^a^
Baseline APACHE II score	27 (24, 31)	26 (22, 30)	0.035^a^
Baseline ISS score	34 (29, 41)	28 (19, 37)	0.011^a^
Blood median storage days	20 (14, 26)	20 (14, 27)	0.651^a^
Blood maximum storage days	25 (19, 32)	25 (19, 31)	0.583^a^
Number of units transfused	4 (2, 6)	4 (2, 9)	0.465^a^

Data given as median (25^th^ percentile, 75^th^ percentile) or percent (frequency) of patients.

^a^Wilcoxon test; ^b^Pearson test.

In the trauma study population, 80 (45%) patients were diagnosed with ALI/ARDS in the 4 days following transfusion. The median storage duration of RBCs transfused in the patients who developed ALI/ARDS was not significantly longer (20 days, IQR 14–26) than the storage duration in patients who did not develop ALI/ARDS (20 days, IQR 14–27, *p* = 0.65). After controlling for age, APACHE II score on enrollment, ISS score on enrollment, smoking status, and number of units transfused, there was no increased risk of developing ALI/ARDS with exposure to blood of a longer median storage duration (OR 0.84 for the 75^th^ vs. 25^th^ percentile of storage duration, CI 0.52-1.35, *p* = 0.46) nor with blood of a longer maximum storage duration (OR 1.05 for the 75^th^ vs. 25^th^ percentile of storage duration, CI 0.72-1.83, *p* = 0.57).

### Nonsepsis, nontrauma patients

Of the 433 patients enrolled who had RBC transfusions during the VALID study, 125 patients were admitted to an ICU for reasons other than sepsis or trauma, which defined the study population for this secondary analysis (Table 5).

**Table 5:** Baseline characteristics of the nonsepsis, nontrauma cohort

**Characteristic**	**ALI/ARDS**	**No ALI/ARDS**	***p *value**
	**(n = 13)**	**(n = 112)**	
Age (yr)	56 (48, 59)	61 (49.8, 68)	0.191^a^
Female gender	69.2% (9)	43.8% (49)	0.081^b^
Current smoker	30.8% (4)	24.3% (27)	0.612^b^
ICU days	6 (3, 8)	5 (3, 9)	0.827^a^
Ventilator-free days	24 (16, 26)	26 (23, 27)	0.094^a^
Hospital days	16 (11, 21)	11 (7, 20.3)	0.337^a^
Baseline APACHE II score	26 (24, 29)	26 (22, 31)	0.711^a^
Blood median storage days	20 (17, 29)	22 (16, 29)	1^a^
Blood maximum storage days	26 (19, 35)	26.5 (20, 33)	0.907^a^
Number of units transfused	4 (2, 5)	4 (2, 8)	0.88^a^

Data given as median (25^th^ percentile, 75^th^ percentile) or percent (frequency) of patients.

N is the number of nonmissing values.

^a^Wilcoxon test; ^b^Pearson test.

In the nonsepsis, nontrauma study population, 13 (10.4%) patients were diagnosed with ALI/ARDS in the 4 days following transfusion. The median storage duration of RBCs transfused in the patients who developed ALI/ARDS was not significantly longer (20 days, IQR 17–29) than the storage duration in patients who did not develop ALI/ARDS (22 days, IQR 16–29, *p* = 1.0). After controlling for age, APACHE II score on enrollment, smoking status, and number of units transfused, there was no increased risk of developing ALI/ARDS with exposure to blood of a longer median storage duration (OR 0.94 for the 75^th^ vs. 25^th^ percentile of storage duration, CI 0.36-2.42, *p* = 0.9) nor with blood of a longer maximum storage duration (OR 1.07 for the 75^th^ vs. 25^th^ percentile of storage duration, CI 0.45-2.51, *p* = 0.88).

## Discussion

In this retrospective, observational study, there was a significant association between the storage duration of RBCs transfused to patients early in the course of sepsis and the risk of developing ALI/ARDS up to 4 days after exposure that was independent of the amount of blood transfused. This association was not seen across other critically ill patient populations, including patients with severe traumatic injuries.

Although several reports have described an increased risk of ALI/ARDS with transfusion of RBCs to critically ill patients that is amplified by the number of units of RBCs transfused [7-11], the current study is unique in that it describes an increased risk of ALI/ARDS in humans with increasing storage duration of blood that is independent of the number of units transfused. In rats and sheep exposed to a “first-hit” (lipopolysaccharide, LPS) as a model of clinical sepsis, transfusion of blood of a longer storage duration caused more severe lung inflammation and ALI compared with fresh blood [27-29,41]. Increased lung inflammation and injury were only observed in those animals that received the dual insult of LPS and blood of a longer storage duration; animals that were transfused with blood of a longer storage duration without LPS exposure had similar rates of ALI compared with controls. Concordant with these animal studies, in the current study, transfusion with blood of a longer storage duration was only associated with ALI in patients with severe sepsis; the association was not seen in severe trauma or critically ill patients who had neither trauma nor sepsis. We did not find an association between the number of units transfused and ALI as others have reported [7-11], which may be explained by our patient population receiving fewer units of blood (median of 2 units RBCs per patient).

Transfusion-related acute lung injury (TRALI) is strictly defined as the onset of ALI/ARDS within 6 hours of a blood transfusion that is not explained by another risk factor [30]. In the current study, we aimed to capture all ALI/ARDS events occurring after transfusion, not just ALI/ARDS that met the strict TRALI definition. We hypothesized that injurious mediators in stored blood might potentiate acute lung injury in the setting of other mediators of acute lung injury, such as sepsis and that this potentiation would not be limited to a 6-hour window after transfusion. For this reason, we had a 48-hour exposure window and included cases of ALI/ARDS occurring as long as 4 days after the exposure, although the majority (88.5%) of cases occurred in the 24 hours after enrollment. Our findings suggest that the risk of ALI/ARDS associated with transfusion in patients with sepsis may continue beyond the 6-hour window that is used to define TRALI [37]. The numerous changes that occur during the storage of RBCs [38] add biologic plausibility to the observation that the potential for poor outcomes is not limited to 6 hours after transfusion.

The current study has several limitations. The retrospective observational design can only suggest association rather than causation. Second, because the vast majority of sepsis patients who developed ALI/ARDS met the criteria for ALI/ARDS at the time of enrollment into the VALID study on the morning of ICU day 2, we used a fixed exposure period of 48 hours before enrollment for ascertainment of RBC exposure; however, there were six patients in the sepsis group who did not develop ALI/ARDS until the days subsequent to enrollment and some of these patients received transfusions beyond the exposure period. As these units of RBCs were unaccounted for in our primary analysis and to ensure these patients were not significantly influencing our results, we repeated the analysis without these patients and still found a significant association. We only studied patients in VALID who had received RBCs before enrollment, which limited our sample size to 96 sepsis patients, 176 trauma patients, and 125 nonsepsis nontrauma patients. Additionally, in the nonsepsis nontrauma subset, there were only 13 patients who developed ALI/ARDS, which significantly limits the power to say anything definitive about this group of patients. Nonetheless, the association between storage duration and ALI/ARDS in patients with sepsis was robust across multiple analyses. Finally, the difference in our study in storage duration of RBCs transfused to patients with sepsis was only 4 days and included transfusion of RBCs of varying age to the same patient, whereas previously published work has focused on transfusion of RBCs of a homogenous storage duration and had greater differences in the storage duration between groups [24,42]. The relatively small difference in storage duration in our study may suggest a less biologically meaningful finding; however, this may rather suggest that even small differences in storage duration are meaningful when transfusing critically ill patients.

Our findings are not concordant with the results of a randomized trial comparing transfusion of fresh blood to blood of longer storage duration in critically ill patients, which showed no difference in the development of ALI between groups [42]. The disparity in results could be due in part to that study being underpowered to detect a difference in this secondary outcome, a problem that might be overcome by a larger, randomized trial currently underway [43]. In addition, animal models and our current findings suggest that signals for increased risk of ALI/ARDS after transfusion may be more robust in septic patient populations rather than heterogeneous, critically ill patient populations as studied in this randomized trial that included only 6 (6%) patients with sepsis. Although not focused on the development of ALI/ARDS, past studies of trauma patients [7,22,25,44,45] showed that the storage duration of RBCs was associated with worse clinical outcomes, which is in contrast to our results. This difference may potentially be explained by a markedly higher median number of RBCs received in the past studies (12 units per patient) [22] compared with our current study (median 4 units per patient in the trauma group). Finally, there is existing data in trauma patients and patients having undergone coronary artery bypass grafting that potential signs of ALI/ARDS (respiratory insufficiency, increased time on the ventilator and pneumonia) more often were found in patients receiving blood of a longer storage duration [24,45]; however, the presence of ALI/ARDS in these patients was not reported.

Our study has potential implications for future research. There was a significant association not only with the median storage duration of transfused blood but also with the maximum storage duration of all units transfused. These findings suggest that there might be increased risk of morbidity and mortality with exposure to even a single unit of RBCs with long storage duration. The current study adds ALI/ARDS to a list of morbidities associated with increasing storage duration of transfused RBCs. Research focusing on the mechanisms that drive the storage lesion and its end-organ effects will provide us with targets for preventative therapy in patients unable to avoid transfusion. Larger, prospective studies are needed to determine which patients (sepsis, trauma, etc.) who required transfusion are at highest risk from aged blood and what threshold delineates hazardous storage duration. Only with additional prospective clinical studies will we be able to create evidenced-based guidelines for transfusion to limit both transfusion rates and transfusion-related morbidity, such as ALI/ARDS.

## Conclusions

Transfusion of blood with longer median storage duration to patients with early sepsis is associated with a higher risk of developing ALI up to 4 days after transfusion, whereas this same association is not seen in patients with trauma or critically ill patients without sepsis or trauma. This may suggest that the storage duration of RBCs transfused to critically ill patients may have variable effects depending on the underlying cause of a patient’s illness.

## Abbreviations

ICU: Intensive Care Unit; RBCs: Packed red blood cells; ALI: Acute lung injury; ARDS: Acute Respiratory Distress Syndrome; VALID: Validating acute lung injury markers for diagnosis; IQR: Interquartile range; APACHE: Acute physiology and chronic health evaluation; OR: Odds ratio.

## Competing interests

The authors declare that they have no competing interests.

## Authors' contributions

DRJ, JAB, PPY, and LBW were involved in the study design. DRJ, BC, AKM, GRB, and PPY collected the data. DRJ, TK, ZZ, and LBW performed the statistical analysis. DRJ drafted the manuscript and all authors participated in the revision of this manuscript. All authors read and approved the final manuscript.
